# Low-dose IL-2 expands CD4^+^ regulatory T cells with a suppressive function *in vitro via* the STAT5-dependent pathway in patients with chronic kidney diseases

**DOI:** 10.1080/0886022X.2018.1456462

**Published:** 2018-04-05

**Authors:** Yuanyuan Li, Xueyong Liu, Wei Wang, Shaohua Wang, Jianchun Zhang, Song Jiang, Yang Wang, Liping Li, Jinghua Li, Youkang Zhang, Haichang Huang

**Affiliations:** a The Kidney Disease Research Center, Jingdong Yumei Kidney Disease Hospital, Beijing, China;; b Renal Division, Key Laboratory of Renal Disease, Department of Medicine, Peking University First Hospital, Beijing, China

**Keywords:** Chronic kidney disease, glomerulonephritis, cytokines, lymphocytes, signaling

## Abstract

**Background:** Patients with chronic kidney disease (CKD) often have CD4+ regulatory T cells (Tregs) dysfunction and chronic inflammation. We aim to investigate the effect, function, and related mechanism of low-dose IL-2 on CD4^+^ regulatory T cells expansion *in vitro* from patients with CKD.

**Methods:** A total of 148 newly diagnosed patients with CKD at Stage III and 35 healthy volunteer subjects were recruited into our studies. The number of peripheral Tregs in peripheral blood mononuclear cells isolated from CKD patients, which were characterized by FACS as CD4^+^CD25^hi^ and CD4^+^CD25^+^FoxP3^+^. The effect of low-dose IL-2 on expansion of Tregs, and the suppressive function of expanded Tregs were also analyzed by FACS. The levels of FoxP3 mRNA were detected by qRT-PCR. The activation of IL-2 induced Stat5 and blocking experiments were assessed by Western Blotting and FACS.

**Results:** We found that the frequency of peripheral Tregs from CKD patients was significantly lower than that in healthy volunteer subjects. We also showed that IL-2 selectively expanded CD4^+^CD25^hi^ and CD4^+^CD25^+^FoxP3^+^ regulatory T cells, and also upregulated the expression of FoxP3 mRNA. Our *in vitro* studies demonstrated that expanded CD4^+^ regulatory T cells from CKD patients suppressed proinflammatory Th1 and Th17 cell response. Furthermore, STAT5 activation is required for IL-2-induced expansion of regulatory T cells and expression of FoxP3 mRNA from CKD patients.

**Conclusions:** Our findings support the clinical Treg defects in CKD patients with glomerular diseases, and the rationale of evaluating low-dose IL-2 treatment for selectively modulating CD4^+^ Tregs.

## Introduction

Chronic and persistent inflammation has been recently recognized as an essential component of chronic kidney disease (CKD), it is also accountable in part for the leading causes of cardiovascular and all-cause mortality [1]. The progression of renal injury usually involves a mixture of innate and cognate processes, and both the immune responses are involved not only in kidney disease activation but also in disease regulation and progression [2]. The CD4^+^ regulatory T cells (Tregs) comprise a small proportion of the total lymphocyte population but can regulate key immune responses across various autoimmune disease settings. Tregs, which specifically express the transcription factor forkhead box P3 (FoxP3), are critical for the maintenance of self-tolerance and play an important role in controlling immune responses of effector T-cells to auto and foreign antigens; they prevent a wide range of clinical conditions such as organ-specific autoimmune diseases, and control anti-tumor responses, anti-viral responses, and immune responses to alloantigens in the setting of organ transplantation [3,4]. They also play an important role in murine models of immune-mediated renal diseases and human glomerular diseases. Reduced suppressive function and the number of peripheral Tregs have been previously reported in autoimmune disorders including several glomerulonephropathy [5–8]. However, the function of peripheral Tregs in patients with CKD has not been clarified.

Interleukin-2 (IL-2) is a crucial cytokine for the function, homeostasis, and survival of CD4^+^CD25^+^FoxP3^+^ regulatory T cells [9]. Currently, it is widely accepted that IL-2 plays a critical role mainly in Treg fitness and homeostasis, partially due to the constitutively high expression of CD25, which makes these cells highly IL-2-dependent. Many recent studies have revealed that low-dose IL-2 therapy preferentially expands CD4^+^ regulatory T cell populations in humans; the results from these studies showed that administration of low-dose IL-2 to patients with chronic graft-versus-host disease (GVHD) [10], autoimmune vasculitis induced by hepatitis C virus infection [11], type I diabetes mellitus [12], and systemic lupus erythematosus [13,14] increased the proportions of functional Tregs. Although not all patients showed clinical improvement, the results are promising. Several approaches have been taken to expand the number of Tregs *in vivo* and *in vitro*, including cytokines such as IL-2 [15] and gene transfer (Foxp3) approaches [16]. Alternatively, Foxp3^+^ Tregs can also be generated *in vitro* by IL-2, and TGF-β from naïve CD4^+^T cells [17], but little is known about the potential of peripheral Tregs from CKD patients as a source for an *in vitro* generated therapeutic cell product.

In this study, we investigated whether low-dose IL-2 could expand CD4^+^CD25^+^Foxp3^+^ regulatory T cells in isolated peripheral blood mononuclear cells from patients with CKD, and the role of the signal transducer and activator of transcription 5 (STAT5) pathway in the expansion. We also tested if expanded Tregs exhibit suppressive functions *in vitro*.

## Materials and methods

### Study patients and healthy volunteer

A total of 148 newly diagnosed as chronic glomerulonephritis and untreated patients with CKD Stage III based on the 2012 classification [18] were recruited and included in the study. All the patients were admitted to the Jingdong Yumei Kidney Disease Hospital between July 2014 and December 2016. Exclusion criteria included patients younger than 18 years of age, pregnant women, and clinical evidence of anemia and other infections. As controls, 35 sex- and age-matched healthy volunteer subjects with no clinical diagnosis of CKD were enrolled in the study. Written informed consent was obtained from all subjects in this study, and all experimental protocols were approved by the Clinical and Research Ethics Committee at Jingdong Yumei Kidney Disease Hospital in Beijing, China.

### Surface and intracellular antigen staining and flow cytometry

PBMCs were isolated by density centrifugation using Histopaque-1077 (Sigma-Aldrich, St. Louis, MO) gradient centrifugation and were then cultured. Cell surface staining was followed by incubation for 30 min on ice with CD4-FITC (BD Biosciences, San Jose, CA), and CD25PE (BD Biosciences). Intracellular cytoplasmic and nuclear antigen staining was performed using the eBiosciences FACS staining protocol, as it allows the simultaneous analysis of cell surface molecules and intracellular antigens at the single-cell level. After surface staining, fixation was followed by permeabilization, and all intracellular staining was performed using the Intracellular Fixation & Permeabilization Buffer Set (eBioscience, San Diego, CA) for the detection of cytoplasmic cytokines IFN-γ and IL-17A. For the detection of nuclear protein FoxP3, the Foxp3/Transcription Factor Buffer Set (eBioscience) was used for nuclear staining. Appropriate isotype controls were included in each experiment.

Flow cytometric analyzes were performed on the FACSCalibur flow cytometer (BD Biosciences) with a total of 20,000 events recorded for each sample through a CD4^+^ lymphocyte gate. Analysis of flow cytometry data was performed using FlowJo software (TreeStar, Inc., Ashland, OR).

### Western blotting

Equal amounts (50 µg) of total protein from treated cell lysates were separated on 10% SDS-PAGE gels. The proteins were electrophoretically transferred to a nitrocellulose membrane. After blocking in 5% nonfat milk for 1 h at room temperature, the membranes were then incubated overnight at 4 °C with various primary antibodies against phospho-Stat5, Stat5 (all at 1:1000), and β-actin (1:5000). Following an extensive washing in PBS containing 0.1% Tween-20, the membranes were then incubated with a horseradish peroxidase-conjugated secondary antibody (anti-mouse at 1: 3000, anti-rabbit at 1: 3000) (Santa Cruz Biotechnology, Santa Cruz, CA) for 1 h at room temperature in PBS. Membranes were then washed with PBS containing 0.1% Tween-20, and the results were obtained using the enhanced chemiluminescence system (PerkinElmer Inc., Boston, MA). After blotting of phospho-Stat5, the same membranes were stripped and reprobed with the anti-Stat5 antibodies and anti-β-Actin, respectively.

### Quantitative RT-PCR

Cells were treated as described for the pimozide inhibit experiment. The cells were collected and lysed in Trizol buffer (Life Technologies). Total RNA was extracted using the RNeasy mini kit (Qiagen, Valencia, CA), and the concentration and purity of total RNA were measured by a Nanodrop 200. The levels of FoxP3 mRNA were determined by quantitative real-time PCR (qRT-PCR) using Qiagen OneStep RT-PCR Kit according to the manufacturer’s protocol. The primers used were: forward primer: 5′-GAA ACAG CAC ATT CCC AGA GTT C-3′; reverse primer: 5′-ATG GCC CAG CGG ATG AG-3′.

### Mixed lymphocyte reactions

We examined the effect of Tregs from CKD patients, which had been exposure to 50 unit/mL of IL-2 for 4 days on the synthesis of cytokines of Th1 and Th17 lymphocytes in mixed lymphocyte reactions (MLR) isolated from human PBMCs. The PBMCs isolated by density centrifugation from healthy donors were used as effector cells and were labeled with 10 µM CFSE (Life Technologies). Then, the CFSE-labeled cells were mixed and cocultured with the expanded CD4+ Tregs at 5:1 from CKD patients for 24 h. CFSE-labeled cells without coculture with Tregs were used as controls. At the last 4 h, phorbol myristate acetate (PMA) (50 ng/ml, Sigma-Aldrich) and ionomycin (500 ng/ml, Sigma-Aldrich) were added. In the last 2 h, Brefeldin A (Sigma-Aldrich) was added. The cells were collected at the end of incubation, and cell surface antigens were stained with anti-CD4-PerCP-CY5.5 (BD Biosciences). The cells were then subjected to intracellular staining with IL-17A-PE and IFN-γ-APC (BD Biosciences). The stained cells were analyzed by flow cytometry.

### Statistics

Data are presented as mean ± SEM from at least three independent experiments. The significance of differences between the comparative groups was analyzed using Student's *t*-test and one-way ANOVA analysis with SigmaStat3.5 software (Chicago, IL). Statistical significance was defined at an alpha value of *p* ≤ .05.

## Results

### The frequency of CD4^+^ CD25^+^ FoxP3^+^ regulatory T cells in PBMCs from CKD patients and normal subjects

**Figure 1.**
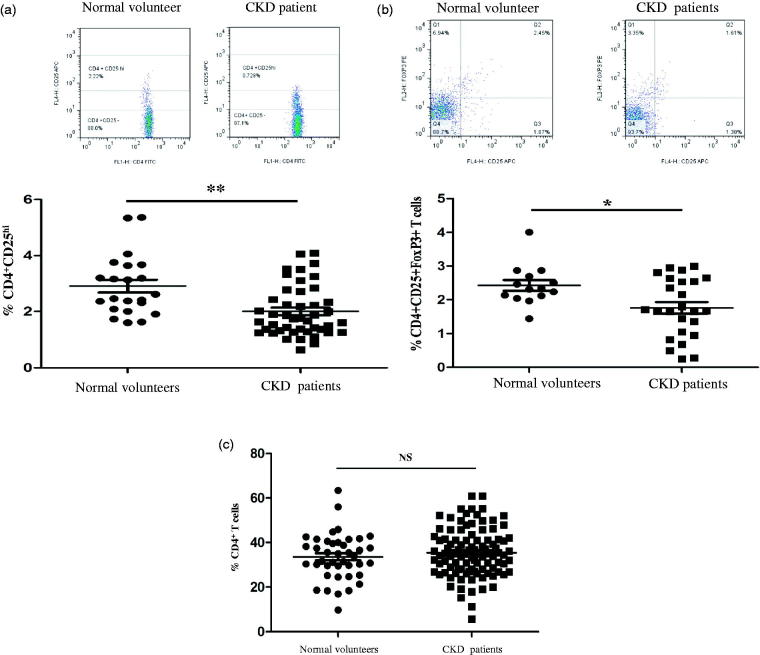
The frequency of peripheral regulatory CD4^+^ T cells (Tregs) in PBMCs from normal subjects and patients with CKD. Tregs were determined within fresh isolated PBMCs of healthy individuals (*n* = 35) and chronic kidney disease (CKD) patients not receiving steroids and immunosuppressant therapy (*n* = 77). (a) A typical example dot plot of the gating strategy CD4^+^CD25^hi^ for Tregs by flow cytometry-based cell characterizing is given (upper panel). In the lower panel, the frequencies of Tregs within CD4^+^ T cells are depicted in a graph. (b) A typical example dot plot of the gating strategy CD4^+^CD25^+^ FoxP3^+^ for Tregs by flow cytometry-based cell characterizing is given (upper panel). In the lower panel, the frequencies of Tregs within CD4^+^ T cells are depicted in a graph. *p* Values are depicted in the graph. (c) The frequencies of total CD4^+^ T lymphocytes are depicted in the graph. *p* Values are depicted in the graph. Study groups were compared using the nonparametric Student’s *t*-test. *p < .05, **p < .01, NS: no significance, for comparisons between the CKD patients group and the healthy controls using *t*-tests.

We at first examined the frequency of CD4^+^ regulatory cells by quantization of CD4^+^CD25^hi^ and CD4^+^CD25^+^Foxp3^+^ in freshly isolated PBMCs from patients with CKD patients with chronic GN and healthy individuals, and the cells were analyzed by flow cytometry. As shown in Figure 1, the frequencies of regulatory T cells from CKD patients that were identified as CD4^+^CD25^hi^ (Figure 1(a)) and CD4^+^CD25^+^FoxP3^+^ (Figure 1(b)) were significantly lower than those in normal individuals. However, the percentages of lymphocytes and CD4^+^ T cells in the PBMCs of CKD patients were comparable to those of normal subjects, as shown in Figure 1(c). Our data indicate for the first that the numbers of CD4^+^ regulatory T cells from PBMCs of CKD patients declined, even though the percentages of lymphocytes and CD4^+^ T cells were sustained at the same levels like that in PBMCs from normal subjects.

### Low-dose IL-2 specifically expands CD4^+^ regulatory T cells in PBMCs from CKD patients

**Figure 2.**
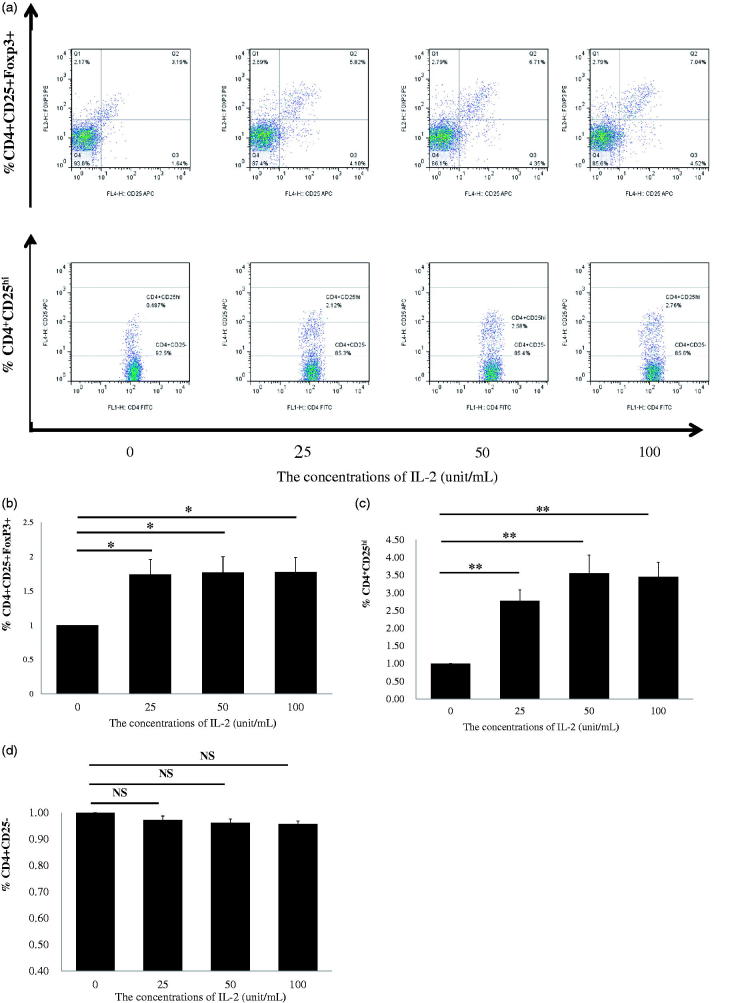
Regulatory T cells (Tregs) of healthy individuals and CKD patients were expanded using PBMCs by rhIL-2 for 4 days. (a) A typical example dot plot of the gating strategy CD4^+^CD25^+^Foxp3^+^ and CD4^+^CD25^hi^ for Tregs for doses of IL-2 at 25, 50, and 100 IU/ml. (b) The frequencies of expanded CD4^+^CD25^+^Foxp3 Tregs within CD4^+^ T cells are depicted in a graph (*n* = 7). (c) The frequencies of CD4^+^CD25^hi^ Tregs within CD4^+^ T cells are depicted in a graph (*n* = 11). (d) The frequencies of CD4^+^CD25^−^ conventional effector CD4^+^ T cells are depicted in a graph (*n* = 18). *p* Values are depicted in the graph. *p < .05, **p < .01 for comparisons between the CKD patients group and healthy controls using one-way ANOVA.

Most recently, low-dose IL-2 has also been shown to expand or modify CD4^+^ regulatory T cells *in vitro* from patients with SLE [13,14] and children with the drug-resistant idiopathic nephrotic syndrome [19]. We aimed to confirm whether low-dose recombinant human IL-2 (rhIL-2) can expand Tregs *in vitro* in PBMCs from CKD patients. The dosages of rhIL-2 at 25, 50, and 100 IU/ml were chosen according to a previous report [9], in which IL-2 concentration <100 IU/ml was considered to be low for the *in vitro* study. PBMCs from 28 patients with CKD were randomly divided into four groups treated with rhIL-2 at 0, 25, 50 and 100 IU/ml concentrations for 4 days, respectively. After 4 days, the PBMCs were collected, and CD4^+^CD25^+^Foxp3^+^ Tregs were analyzed by FACS (Figure 2(a)). Compared with the untreated control (0 IU/ml) group of PBMCs, IL-2 treatments at 25, 50 and 100 IU/ml significantly induced expansion of CD4^+^CD25^+^Foxp3^+^ Tregs, and ∼70% more Tregs were expanded by all three doses of IL-2 compared with untreated cells (Figure 2(b)). Because the volume of venous blood for examination of FoxP3 in PBMCs from CKD patients was limited, we collected additional blood samples from 44 patients with CKD, which were randomly divided into four groups. The isolated PBMCs were treated with IL-2 at 0, 25, 50 and 100 IU/ml for 4 days, respectively, and the percentage of CD4^+^CD25^hi^ Tregs was analyzed. As shown in Figure 2(c), CD4^+^CD25^hi^ Treg expansion was induced by IL-2 as compared with untreated cells. Furthermore, the effects of rhIL-2 on Treg expansion were dose-dependent; nearly 250% more Tregs were expanded by 50 and 100 IU/ml of IL-2 compared with the untreated cells. We also confirmed the same findings as in previous reports [13,14] that low-dose IL-2 treatment did not expand CD4^+^CD25-effector T cells in PBMCs from CKD patients (Figure 2(d)). Together, these results show for the first time that low-dose IL-2 treatment in PBMCs from CKD patients specifically expands Tregs *in vitro*, and these results imply that low-dose IL-2 administration could modify endogenous peripheral Tregs from CKD patients.

### Low-dose IL-2-expanded regulatory T cells from CKD patients suppress the production of Th1 and Th17 cytokines in PBMCs

**Figure 3.**
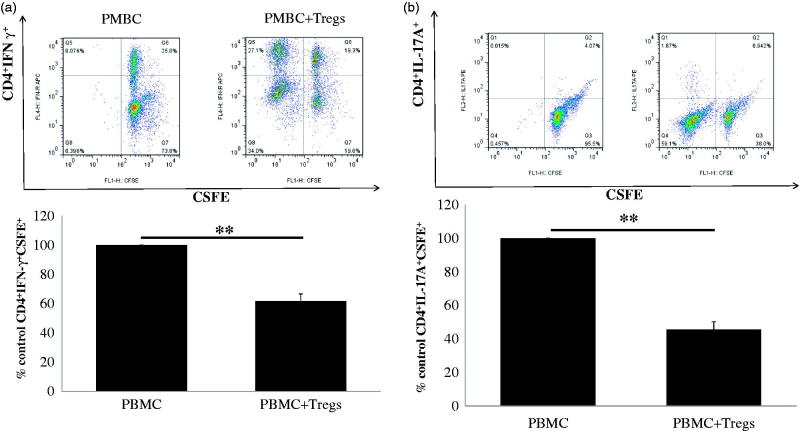
Suppressive capacity of allogeneic low-dose IL-2-expanded regulatory T cells (Tregs). Suppressive capacity of IL-2-expanded Tregs obtained from 17 CKD patients was tested in a mixed lymphocyte reaction, inducing IFN-γ-producing Th1 cells and IL-17A-producing Th17 cells to the initial stimulus (i.e. alloantigen-induced proliferation. (a) A typical example dot plot of the gating strategy CSFE^+^ IFN-γ^+^ for Th1 cells by flow cytometry-based cell characterizing is given (upper panel). In the lower panel, a typical example of the percentage of inhibition on IFN-γ-producing Th1 cells is depicted (*n* = 17). (b) A typical example dot plot of the gating strategy CSFE^+^ IL-17A^+^ for Th17 cells by flow cytometry-based cell characterizing is given (upper panel). In the lower panel, a typical example of the percentage of inhibition on IFN-γ-producing Th1 cells is depicted in the graph (*n* = 17). *p* Values are depicted in the graph. *p < .05, **p < .01 for comparisons between the IL-2 treated group and the non-IL-2 treated group using *t*-tests.

Multiple studies have identified CD4^+^ helper T cells, Th1 and Th17, as the central players of GN. Th1 and Th17 responses cause renal tissue damage, while Tregs mediate protection [20]. This prompted us to ask whether the low-dose IL-2-expanded regulatory T cells have a suppressive function on the production of cytokines of effector Th1 and Th17 cells *in vitro*. As shown in Figure 3, when PBMCs from normal subjects were cocultured with expanded CD4^+^CD25^high^ regulatory T cells from CKD patients, a significant decrease in the percentage of IFN-γ^+^ Th1 (Figure 3(a)) and IL-17A^+^ Th17 cells (Figure 3(b)) in normal PBMCs was observed compared with no coculture with Tregs. Our results suggest that *ex vivo*-expanded Tregs exhibit suppressive function in the production of Th1 and Th17 cytokines *in vitro*. Taken together, our data strongly support the possibility of using *ex vivo*-expanded natural Tregs as a cellular therapy for CKD patients with chronic GN.

### Low-dose IL-2 alter the expression of Foxp3 mRNA in stimulated PBMCs

**Figure 4.**
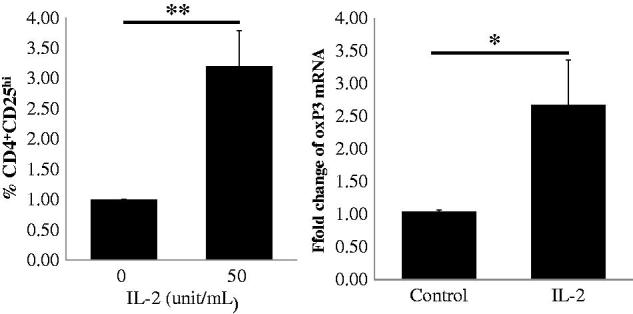
Expression of FoxP3 mRNA in CD4^+^CD25^hi^ Tregs in PBMCs from 10 CKD patients following low-dose IL-2 treatment. In the upper panel, CD4^+^CD25^hi^ Tregs expansion in PBMCs from CKD patients induced by IL-2 is depicted in the graph (*n* = 10). The expression level of FoxP3 mRNA in the IL-2 treated PMBCs is depicted in the graph (*n* = 10). *p* Values are depicted in the graph. *p < .05, **p < .01 for comparisons between the IL-2 treated group and the non-IL-2 treated group using *t*-tests.

Because the transcription factor FoxP3 is the master regulator for the regulatory T cell lineage and levels of Foxp3 expression have been interpreted primarily to reflect Treg frequency [21], we further analyzed the Foxp3 mRNA levels of IL-2-expanded Tregs. In this study, we chose a dose of 50 IU/ml IL-2 to stimulate PBMCs from 10 patients for 4 days and detected the frequency of CD4^+^CD25^hi^ Tregs by FACS and mRNA expression of FoxP3 by real-time qRT-PCR. We found that the levels of FoxP3 mRNA increased significantly compared to the no IL-2 stimulation control, as illustrated in Figure 4, and the magnitude of increase in FoxP3 mRNA levels was similar to the increase in the frequency of CD4^+^CD25^hi^ Tregs. Our data indicate that low-dose IL-2 could stimulate the production of Tregs *ex vivo* by inducing FoxP3 mRNA expression.

### STAT5 phosphorylation is required for low-dose IL-2-induced expansion of CD4^+^ regulatory T cells and expression of FoxP3 mRNA in PBMCs

**Figure 5.**
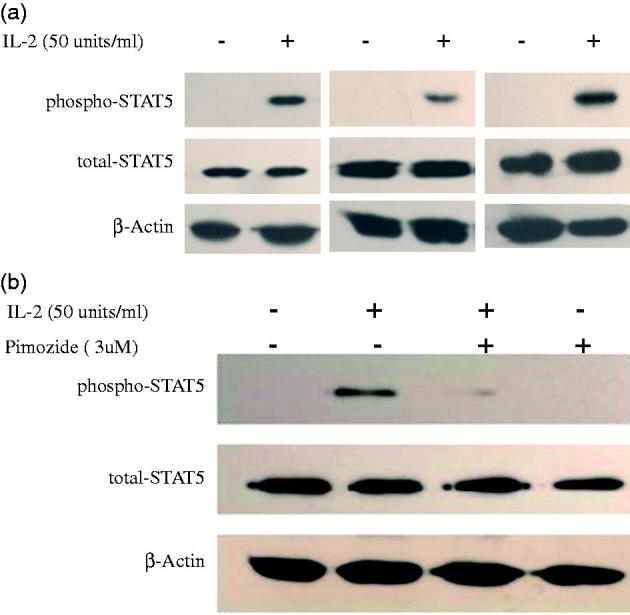
Phosphorylation of STAT5 in expanded Tregs in PBMCs from CKD patients by low-dose IL-2. (a) Three representative blots of Western blot analysis for phospho-STAT5 in PBMCs from CKD patients induced by IL-2 is depicted in the graph. (b) A representative blot of Western blot analysis for pimozide, a specific inhibitor of STAT5 activation; abolishment of IL-2-induced STAT5 phosphorylation is depicted in the graph.

Several studies have supported a role of STAT5 signaling in IL2-induced regulatory T cell development from patients with different illness [22,23]. However, the role of STAT5 in regulatory T cell production in CKD patients is unknown. We investigated the effect of low-dose IL-2 on the phosphorylation of STAT5 in expanded Tregs from CKD patients. As shown in Figure 5(a), IL-2 remarkably stimulated STAT5 phosphorylation in PBMCs from CKD patients. Pimozide is a specific inhibitor of STAT5 phosphorylation, and we examined its effect on STAT5 phosphorylation by pretreating cells with it. As seen in Figure 5(b), 3 µM pimozide pretreatment dramatically suppressed IL-2-induced STAT5 phosphorylation, indicating that it is a potent blocker of IL-2-stimulated STAT5 phosphorylation in PBMCs from CKD patients.

**Figure 6.**
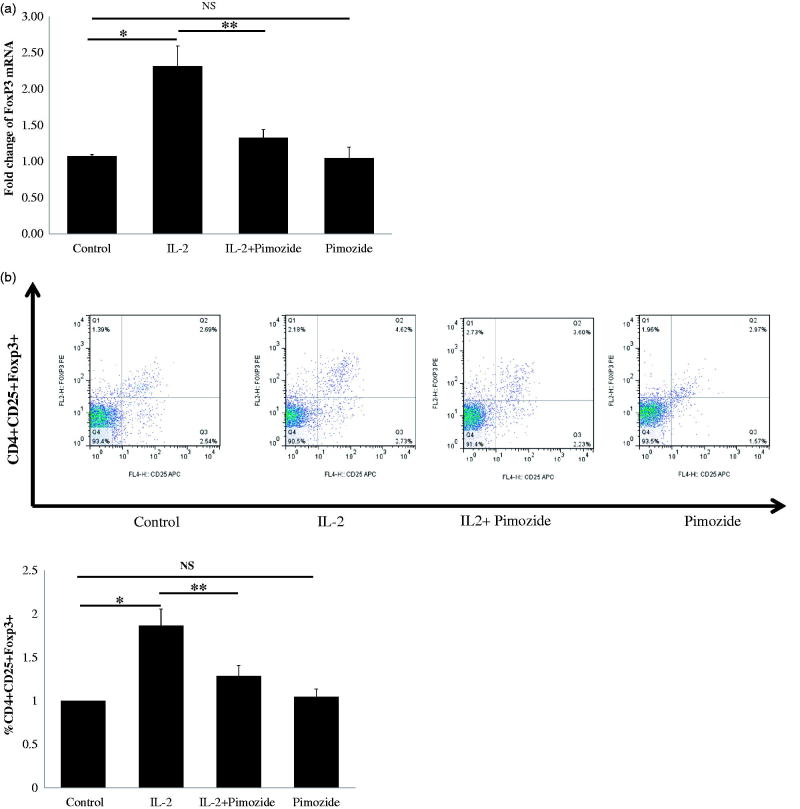
Blockage of STAT5 activation attenuates IL-2-induced Tregs expansion and FoxP3 mRNA expression in PBMCs from 10 CKD patients. (a) A typical example dot plot of the gating strategy CD4^+^CD25^+^ FoxP3^+^ and CD4^+^CD25^hi^ for Tregs by flow cytometry-based cell characterizing is given. (b) Block of activation of STAT5 by pimozide-attenuated IL-2-induced CD4^+^CD25^+^ FoxP3^+^ Tregs expansion is depicted in the graph (*n* = 9). The data are expressed as the mean ± SEM, with nine patients per group. (c) Block of activation of STAT5 by pimozide-attenuated IL-2-induced CD4^+^CD25^hi^ Tregs expansion is depicted in the graph (*n* = 7). The data are expressed as the mean ± SEM. (d) Block of activation of STAT5 by pimozide-attenuated IL-2-induced expression of FoxP3 mRNA is depicted in the graph. The data are expressed as the mean ± SEM. *p* Values are depicted in the graph. *p < .05, **p < .01 for comparisons between the IL-2 treated group and the non-IL-2 treated group using one-way ANOVA analysis.

We further tried to determine whether inhibition of STAT5 activation could abrogate low-dose IL-2-mediated FoxP3 mRNA expression and Treg expansion in PBMCs from patients with CKD. Pimozide pretreatment significantly suppressed IL-2-induced expression levels of FoxP3 mRNA (Figure 6(a)), as well as the markedly decreased IL-2-stimulated expansion of CD4^+^CD25^+^Foxp3^+^ T regulatory cells (Figure 6(b)). These results demonstrate that low-dose IL-2 promotes the generation of CD4^+^CD25^+^Foxp3^+^ T regulatory cells through the STAT5 pathway, and provides additional evidence for supporting the role of IL2 and STAT5 in regulatory T cell development and function.

## Discussion

In this study, we demonstrated that Tregs are normally present in low numbers in CKD patients with chronic glomerular diseases. Other studies have shown both reduced Treg number in patients with lupus nephritis, IgA nephropathy, and in pediatric patients with the nephritic syndrome [5,19,24]. Together, these results suggest that reduction in Treg number and/or function may play a role in immune-mediated glomerular diseases and injury. In this study, we demonstrated that low-dose IL-2 could selectively expand Tregs *ex vivo* in PBMCs isolated from CKD patients, and expanded Tregs exhibit effective and potent suppressive function against the production of Th1 and Th17 cells. A recent study by Litjens et al. [25] provided a feasible and effective isolation and large-scale *ex vivo* expansion of circulating nTregs from CKD patients. This could make Tregs a potential cellular immunotherapy, as an alternative or supplement to current immunosuppressive medication regimes for patients with immune-mediated CKD. *Ex vivo* expansion and transfer modificated Foxp3-transduced Tregs or *in vivo-*induced Tregs can protect against ischemia acute kidney injury (AKI) [26] and decrease infiltration of inflammatory cells and proteinuria in CKD animal models [27]. Our data also documented an important role of STAT5 activation for IL-2 and in shaping the development of peripheral Tregs in patients with CKD, together with other studies have demonstrated that IL-2 and STAT5 play critical roles in maintaining the stability and function of the peripheral Treg lineage [23].

Dysregulated systemic helper T cell Th1 and Th17 immunity was proven to be central to the development of kidney injury in renal inflammatory diseases, initiation of human GN, and animal GN model [20,28]. In contrast to pathogenic Th1 and Th17 responses, regulatory T cells (Tregs) were proven to be inhibitors of various effector T cells such as IFN-γ-producing Th1 cells and the recently identified IL-17^+^ T helper 17 (Th17) cells in GN [29]. In this study, we demonstrate for the first time that *ex vivo* expanded Tregs in PBMCs from CKD patients can suppress IFN-γ-producing Th1 cells and pathogenic IL-17A producing-Th17 cells by responder T cells. Our findings suggest that Treg cells play an important role in constraining pathogenic Th17 cells and in preventing GN. Indeed, we found that there are significant differences in the frequency of total Foxp3^+^ Treg cells between CKD patients and normal control individuals. Recently, human Treg therapy is an attractive option for many immune-mediated kidney diseases and immune injury in transplantation [30]. This new and evolving field of cellular therapy would allow for the use of functional Treg cells that have the capacity to suppress both potentially pathogenic Th1 and Th17 cells and is an attractive option for treating immune-mediated kidneys shortly.

In conclusion, our present study demonstrated for the first time that low-dose IL-2 can expand CD4^+^CD25^+^FoxP3^+^Tregs preferentially *via* the STAT5 pathway in PBMCs from CKD patients with chronic glomerular diseases *in vitro*, and the expanded Tregs exert potential suppressive function on the production of proinflammatory Th1 and Th17 cytokines. Our data implied that low-dose IL-2 therapy has a promising role in the treatment of CKD patients with chronic glomerular diseases as a novel, effective, and safe therapeutic approach.
